# NR3C2 inhibits the proliferation of colorectal cancer via regulating glucose metabolism and phosphorylating AMPK

**DOI:** 10.1111/jcmm.17706

**Published:** 2023-03-23

**Authors:** Hui Liu, Wenqi Lei, Zhigui Li, Xiaodong Wang, Liming Zhou

**Affiliations:** ^1^ Department of Pharmacology, West China School of Basic Science and Forensic Medicine Sichuan University Chengdu China; ^2^ Department of Gastrointestinal Surgery West China Hospital Sichuan University Chengdu China

**Keywords:** AMPK, colorectal cancer, glucose metabolism, NR3C2

## Abstract

We aim to investigate the roles and mechanisms of NR3C2 in colorectal cancer (CRC). The expression of NR3C2 in CRC tumours and paired paracancerous tissues of 71 CRC patients and five CRC cell lines was detected by western blotting, immunohistochemistry and real‐time reverse‐transcription PCR. Moreover, NR3C2 was overexpressed or knocked down in CRC cells by lentiviral vector transfection. The proliferation of cells was measured by MTT, colony formation assay and flow cytometry. Glucose metabolism was assessed by detecting lactate production, glucose consumption and ATP production. Western blotting and real‐time reverse‐transcription PCR were used to detect the expression of AMPK, LDHA and HK2. The expression of NR3C2 was significantly decreased in CRC tumours compared to paracancerous tissues, which was correlated with distant metastasis, poor prognosis and advanced stages of CRC patients. Overexpressing NR3C2 suppressed the proliferation and promoted the G2/M cell cycle arrest of CRC cells. Furthermore, NR3C2 inhibited glucose metabolism by decreasing the expression of HK2 and LDHA. The phosphorylation of AMPK was also downregulated in CRC cells overexpressing NR3C2. This study demonstrated that NR3C2 inhibited the proliferation of CRC by inhibiting glucose metabolism and phosphorylation of AMPK which may serve as a therapeutic target for CRC.

## 1 INTRODUCTION

Colorectal cancer (CRC) is the third most common malignancy diagnosed globally and the second leading cause of cancer‐related death worldwide according to the 2020 Globocan database.^1^ It is estimated that global CRC incident cases will increase from 1.93 million in 2020 to 3.2 million in 2040.^2^ At the time of diagnosis, approximately half of the patients are in a middle or advanced stage, and recurrences and metastases also occur following surgery.^3^ Therefore, it is urgent to identify novel pharmacological targets to improve therapeutic effects and prognosis.

To identify key genes involved in the progression of CRC, we performed differentially expressed genes (DEGs) analysis across five data sets from the Gene Expression Omnibus (GEO) database. Among 72 DEGs, NR3C2 was identified as one of the downregulated genes associated with CRC prognosis. The NR3C2 gene encodes the human mineralocorticoid receptor (hMR), which belongs to the nuclear receptor superfamily and steroid receptor subfamily. hMR is widely distributed in tissues and has numerous functions in cardiovascular function, immune cell signalling, neuronal fate and adipocyte differentiation,^4^ which regulates salt balance and water homeostasis in classical polarized epithelial tissues such as the colon. It has been reported that NR3C2 inhibits the migration, invasion, and epithelial‐to‐mesenchymal transition of pancreatic cancer cells.^5^ NR3C2 has also been shown to inhibit the proliferation and metastasis in SMMC‐7721 and HCCLM3 liver cancer cells, as well as 786‐O and ACHN kidney cancer cells.^6^, ^7^ The expression of NR3C2 was negatively correlated with VEGFR‐2 and inhibited the proliferation and migration of LoVo cells by inhibiting the AKT/ERK pathway.^8^, ^9^, ^10^ Besides, database analysis found that promoter hypermethylation may be the reason for NR3C2 low expression in colon cancer.^11^

AMP‐activated protein kinase (AMPK) is a well‐known energy sensor that plays a key role in sustaining energy homeostasis and is implicated in the development of cancer. It has been demonstrated that AMPK exerts both carcinogenic and anti‐tumorigenic functions in tumours, depending on the context.^12^ The expression of AMPK is increased in CRC and associated with poor prognoses, as well as promoting the migration and invasion of CRC cells when it is activated.^12^, ^13^ So we wanted to explore the role of NR3C2 in AMPK expression and activation in CRC. Tumours reprogram nutrition acquisition and metabolic pathways to fulfil the demands of malignant cells for bioenergetic, biosynthetic and redox functions.^14^ Nie and his colleagues demonstrated that NR3C2 suppresses the progression and Warburg effects of hepatocellular carcinoma.^15^ Besides, the effects of NR3C2 on energy metabolism have been reviewed.^16^, ^17^ However, the underlying mechanism of NR3C2 in the glucose metabolism of CRC remains unclear.

This study aims to investigate the biological function and clinical significance of NR3C2 and the mechanism by which NR3C2 influences the proliferation of CRC. We discovered that overexpression of NR3C2 inhibits tumour progression by suppressing glucose metabolism and AMPK expression.

## 2 MATERIALS AND METHODS

### 2.1 Patients and tissue samples

The 71 cases of primary CRC tissues and paracancerous tissues (5.0 cm beyond the tumour tissues) were collected at the Department of Surgery, West China Hospital, Sichuan University between November 2019 and November 2020. None of the patients underwent chemotherapy before surgical resection. The collection and use of human tissues for this study were approved by the Ethics Committee of West China Hospital. Written informed consent was obtained from patients before sampling.

### 2.2 Microarray data and identification of DEGs

The GEO (http://www.ncbi.nlm.nih.gov/geo) is a freely available public functional genomics data repository. Five gene microarrays including GSE41258, GSE81582, GSE21510, GSE113513 and GSE22598, were identified and downloaded from the GEO database. The microarray data of GSE21510 and GSE22598 were based on the GPL570 platform and collected by Affymetrix Human Genome U133 Plus 2.0 arrays. GSE81582 and GSE113513 were based on the GPL15207 platform and collected by Affymetrix Human Gene Expression Array. GSE41258 was based on the GPL96 platform and collected by Affymetrix Human Genome U133 arrays. A total of 363 CRC samples were included in these five profiles. Then, the GEO2R online tool (https://www.ncbi.nlm.nih.gov/geo/geo2r/) was employed to detect DEGs of primary CRC tissues and paracancerous tissues. *p* < 0.05 and |log fold change (FC)| ≥ 2 were set as the cut‐off criteria. Venn diagram software (http://bioinformatics.psb.ugent.be/webtools/Venn/) was used to identify consistent DEGs. In addition, the difference in the expression of NR3C2 between CRC tissues and paracancerous tissues was also conducted on five gene microarrays.

### 2.3 Survival analysis

The clinical data of 579 patients obtained from GSE39582 were used for survival analysis. The Kaplan–Meier method was performed. A log‐rank test was used to compare the survival distributions, and *p* < 0.05 was considered statistically significant. Survival curves were plotted in GraphPad Prism (version 5, GraphPad, Inc.).

### 2.4 Bioinformatics analysis

The online database Gene Expression Profiling Interactive Analysis (GEPIA, http://gepia.cancer‐pku.cn/index.html) was used to perform survival analysis and detect differential expression of NR3C2 between CRC tissues and paracancerous tissues based on the TCGA data set. Gene set enrichment analysis (GSEA, http://www.broad.mit.edu/gsea) was performed to identify gene sets changed by high NR3C2 expression versus low NR3C2 expression from the microarray data of GSE41258. Tumor Immune Estimation Resource (TIMER, https://cistrome.shinyapps.io/timer/) was used to analyse the expression of NR3C2 in different types of cancer.

### 2.5 Gene expression data analysis

The tumour–node–metastasis (TNM) stage data obtained from GSE39582 and GSE41258 were used to analyse the correlation between the expression of NR3C2 and TNM stages. TCGA data sets were downloaded from Oncomine (http://www.oncomine.org) to analyse the correlation between the expression of NR3C2 and distant metastasis.

### 2.6 Immunohistochemistry (IHC)

The expressions of NR3C2 proteins were determined by IHC in the CRC tumours and paracancerous tissues. All samples were obtained from the West China Hospital Biobank of Sichuan University. The embedding and staining of paraffin blocks were as previously described.^18^ Paraffin‐embedded biopsies were incubated with primary antibodies against NR3C2 (1:100; Abcam). NR3C2 expression was evaluated and quantified using ImageJ with IHC Toolbox.

### 2.7 Cell culture and treatment

Five CRC cell lines, HCT116, RKO, SW480, SW620, DLD‐1, and human 293T embryonic kidney cells were purchased from American Type Culture Collection (ATCC, Manassas, VA, USA). They were cultured in the Dulbecco's modified Eagle's medium (DMEM) supplemented with 10% fetal bovine serum, 100 U/mL penicillin, and 100 μg/mL streptomycin. Cells were cultured in a humidified incubator with 5% CO_2_ at 37°C.

Spirolactone (SPI, Selleck, S4054) and Aldosterone (Ald, Solarbio, IA0700) were diluted to 10 mM and 1 mg/mL with DMSO and ethanol, respectively. HCT116 cells were treated with SPI at 2.5 μM for 1 h, followed by Ald at 0.35 μM for 72 h.

### 2.8 Cell transfection

NR3C2 shRNA and negative control shRNA were commercially obtained from Tsingke (Beijing, China). Sequences of NR3C2 shRNA were as follows: CCGG‐CCAGCTAAGATTTATCAGAAT‐CTCGAG‐ATTCTGATAAATCTTAGCTGG‐TTTTTT. The shRNA was cloned into the lentiviral vector PLK0.1. NR3C2 cDNA was cloned into the lentiviral expression vector GV492 (GeneChem).

Virus packaging was performed in 293T cells after co‐transfection of psPAX2 and pMD2.G vector using Lipofectamine 3000 (Invitrogen). Viruses were harvested at 48 h after transfection, and then infected HCT116, RKO, and DLD‐1 cells with polybrene (10 μg/mL; Sigma‐Aldrich) for 48 h. Fluorescence‐activated cell sorter (BD, FACS Ariall) was used to screen cells with GFP fluorescence which indicated that cells were successfully infected.

### 2.9 Cell proliferation

Transfected cells were seeded into 96‐well plates and cultured for 1–6 days. Then, 100 μL of 3‐(4,5‐dimethyl‐2‐thiazolyl)‐2,5‐diphenyl‐2‐H‐tetrazolium bromide (MTT) dissolved in the medium (0.5 mg/mL) was added to each well. After incubation for 2.5 h, the culture medium was discarded and 150 μL DMSO was added. The plate was gently shaken in darkness for 10 min until the crystal fully dissolved. Absorbance at 490 nm was detected by the microplate reader (MD, SpectraMax 190).

Cells were seeded into 96‐well plates and after being cultured for 24 h; cells were treated with 3 μM Compound C (CC, MCE, HY‐13418), 100 μM AICAR (MCE, HY‐13417A), or 5 mM 2‐DG (Selleck, S4701), respectively. When CC and AICAR were used in combination, cells were treated with 3 μM CC for 15 min before being incubated with 100 μM AICAR. MTT assays were performed after cells were cultured for the indicated days.

### 2.10 Colony formation assay

Cells were seeded into 6‐well plates at a density of 500 cells/well and changed to fresh medium or drug‐containing medium every 3 days for a total of 14 days. The cells were then fixed with paraformaldehyde for 30 min and stained with 0.1% crystal violet. All the visible colonies were counted using ImageJ software.

### 2.11 Real‐time RT‐PCR

Trizol reagent (Invitrogen, USA) was used to obtain the total RNA of tissues and cells according to the instructions of the manufacturer. Concentrations and purity of RNA were determined by Nanodrop One (Thermo Fisher). First‐strand cDNA was synthesized using PrimeScript™ RT reagent Kit with gDNA Eraser (TaKaRa, RR047A,). qPCR analysis was performed with SYBR Green Master Mix (TaKaRa, RR820A) on a CFX96 Touch™ qPCR system (Bio‐Rad) with the following conditions: 95°C for 30 s, 35 cycles of 95°C for 5 s and 61.4 °C for 30 s. The mRNA expression of NR3C2, LDHA, HK2, and GLUT1 was normalized to GAPDH. Data were normalized and expressed as the fold change relative to the control sample. The primers used were as follows: NR3C2 forward primer: 5′‐GAGAGCCCACATTGCTAGCA‐3′, reverse primer: 5′‐GCCCTGCTGGAATCAACTCT‐3′; LDHA forward primer: 5′‐AGCCCGATTCCGTTACCT‐3′, reverse primer: 5′‐CACCAGCAACATTCATTCCA‐3′; GLUT1 forward primer: 5′‐ATTGGCTCCGGTATCGTCAAC‐3′, reverse primer: 5′‐GCTCAGATAGGACATCCAGGGTA‐3′; HK2 forward primer: 5′‐GATTTCACCAAGCGTGGACT‐3′, reverse primer: 5′‐CCACACCCACTGTCACTTTG‐3′; and GAPDH forward primer: 5′‐AGAAGGCTGGGGCTCATTTGC‐3′, reverse primer: 5′‐ACAGTCTTCTGGGTGGCAGTG‐3′.

### 2.12 Western blotting

Western blotting was performed as described previously.^19^ The protein samples were extracted from tissues and cells by RIPA lysis buffer with protease inhibitor PMSF, separated by 10% SDS‐PAGE and transferred to polyvinylidene difluoride membranes (Millipore Corp). The membranes were incubated overnight at 4°C with primary antibodies against NR3C2 (1:600; ab64457, Abcam), AMPKα (1:1000; #5831, Cell Signalling Technology), p‐AMPK(Thr172) (1:1000; #2535; Cell Signalling Technology) LDHA (1:3000; 19,987‐1‐AP, Proteintech), HK2 (1:1000; 66,974‐1‐Ig, Proteintech) or β‐actin (1:3000; TA‐09, ZSGB‐BIO, China). Then, membranes were incubated with the secondary antibody conjugated to HRP (anti‐rabbit IgG, anti‐mouse IgG, ZSGB‐BIO) at room temperature for 2 h. Signal intensity was detcted using ECL Plus reagents (P0018S, Beyotime, China) on chemiluminescence imaging system (ChemiScope 6000, CLiNX, China) and quantitated using ImageJ software.

### 2.13 Flow cytometry

Cells were cultured in 6‐well plates for 48 h, the serum‐free medium was replaced and cultured for 12 h. Then, the complete medium was replaced and the cells were collected after being cultured for another 24 h. After washing with pre‐cold phosphate buffered saline (PBS), the cells were incubated with 95% ethanol at 4°C for 48 h. Then, cells were washed with pre‐cold PBS again and stained with PI (FXP0211,4A biotech, China) according to the instructions of the manufacturer. The cell cycle was analysed by the Millipore Guava Flow cytometer (Millipore Corp, Bedford, MA, USA).

### 2.14 Glucose consumption assay

Cells were seeded into 96‐well plates at a density of 3000/well. After being cultured for 24 h, cells were washed with PBS and incubated with a serum‐free medium overnight. Then, cells were washed with PBS and starved for glucose by pre‐incubating with 100 μL DMEM medium (without glucose) containing 2%BSA for 1 h. DMEM medium with 6 mM glucose containing 10% FBS was added to each well for 18 h. The supernatant was collected and performed MTT assay on cells in 96‐well plates for correction. The glucose concentration of the supernatant was quantified by a glucose consumption assay kit (E1010, Applygen Technologies, China) and data were determined using a microplate reader (MD, SpectraMax 190) at 550 nm.

### 2.15 Ldh activity and lactic acid production assay

Cells were seeded into 96‐well plates at a density of 3000/well. After being cultured for 24 h, cells were cultured with a serum‐free medium for 12 h and incubated with a complete medium for another 24 h. The supernatant was collected and the concentration of lactate was measured according to the instruction of the lactic acid detection kit (A019‐2, Nanjing Jiancheng) and normalized according to the MTT assay. The absorbance was measured using a microplate reader (MD, SpectraMax 190) at 530 nm.

Ldh activity was determined using commercial kits for Ldh activity assay (A020‐2, Nanjing Jiancheng). Protein concentration was used to adjust the cell quantity. The absorbance was measured using a microplate reader (MD, SpectraMax 190) at 450 nm.

### 2.16 ATP measurements

The cells in 6‐well plates were washed three times with PBS, and then 200 μL of ATP extraction solution was added to each well and pipetted repeatedly on ice to lyse the cells. After being centrifuged at 4°C for 5 min, the supernatant was collected to determine ATP content with the ATP Assay kit (S0026, Beyotime, China). Protein concentration was used to adjust the cell quantity. The luminescence was measured using a microplate reader (Tecan, Switzerland).

### 2.17 Statistical analysis

All experiments were performed at least in triplicate. The results were presented with mean ± standard deviation and analysed using Prism 5 (GraphPad Software). Data were analysed by the Student's *t*‐test or one‐way anova. Pearson's chi‐square test was used to analyse the correlation between the clinical characteristics and expression of NR3C2 using IBM SPSS statistic version 21 (SPSS Inc.). The statistical significance was set at *p* < 0.05.

## 3 RESULTS

### 3.1 Identification of DEGs between CRC tumours and paracancerous tissues

**FIGURE 1:**
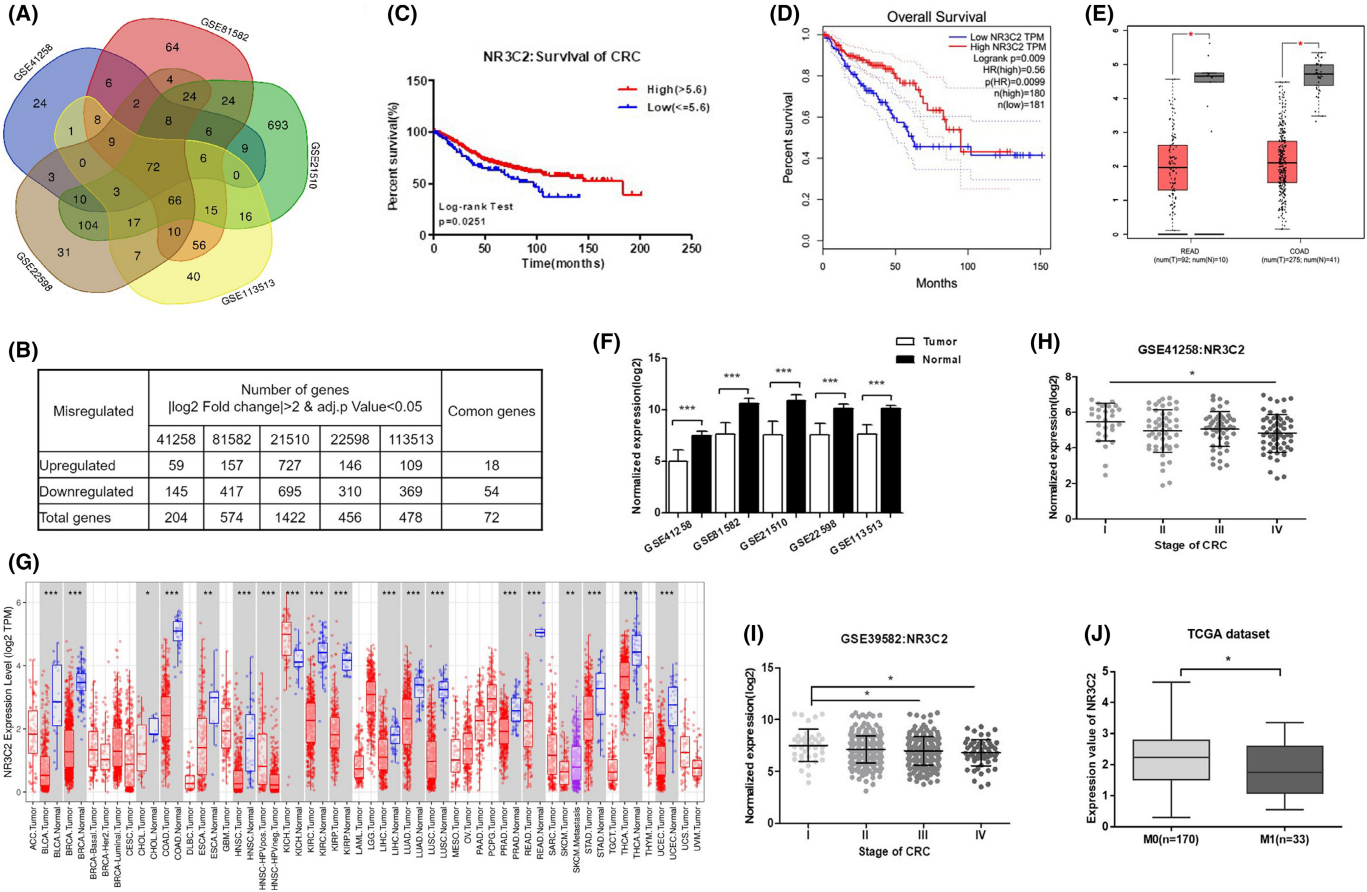
The expression of NR3C2 was downregulated and associated with survival of CRC. (A) Venn diagram analysis of DEGs based on five data sets (GSE41258, GSE81582, GSE21510, GSE113513 and GSE22598). (B) The numbers of upregulated and downregulated genes among DEGs in CRC tumours compared to adjacent normal tissues. (C, D) Overall survival analysis of CRC patients with high or low expression of NR3C2 with data from GSE39582 and GEPIA. (E) Expressions of NR3C2 in COAD and READ samples were analysed by GEPIA. (F) The expressions of NR3C2 in CRC tumours compared to adjacent normal tissues in the five data sets including GSE41258, GSE81582, GSE21510, GSE113513 and GSE22598. (G) The expression levels of NR3C2 in different tumour types from the TCGA database were determined by TIMER (***p < 0.001, **p < 0.01 and *p < 0.05). (H, I) The expression of NR3C2 in CRC tumours from the GSE41258 and GSE39582 data set were analysed based on TNM stages. (J) The expression of NR3C2 in CRC tumours in the TCGA data set was analysed based on CRC distant metastasis. *p < 0.05, ***p < 0.001. COAD, colon adenocarcinoma; CRC, colorectal cancer; DEGs, differentially expressed genes; GEPIA, gene expression profiling interactive analysis; READ, rectum adenocarcinoma; TCGA, the cancer genome atlas.

To identify the key genes involved in the progression of CRC, expressions of DEGs were analysed by GEO2R. Following integrated bioinformatics analysis, a total of 72 consistent DEGs were identified from the five gene expression profile data sets (Figure 1A). Compared with paracancerous tissues, there were 18 upregulated genes and 54 downregulated genes in CRC tumours (Figure 1B).

The overall survival data of 579 patients were obtained from the GSE39582 data set. Survival analyses were performed based on their high or low expressions of NR3C2. High expression of NR3C2 was associated with higher overall survival in CRC patients (Figure 1C). Similar overall survival analysis results were obtained from GEPIA (Figure 1D). We then compared the expression of NR3C2 in CRC tumours and adjacent normal tissues in each of the five data sets. Notably, the expression of NR3C2 was significantly decreased in CRC tumours than in adjacent normal tissues in all five data sets (Figure 1F). The GEPIA database was also used to compare the expression of NR3C2 in CRC tumours and paracancerous tissues. Accordingly, the expression of NR3C2 was lower in both colon cancer and rectum cancer than in paracancerous tissues (Figure 1E). In addition to CRC, NR3C2 was also downregulated in 15 types of cancer tissues such as bladder, breast and kidney (Figure 1G). More importantly, we found that the lower expression of NR3C2 in CRC patients was associated with advanced stages and distant metastasis (Figure 1H–J).

### 3.2 NR3C2 was downregulated in CRC tumours as determined in clinical samples

**FIGURE 2:**
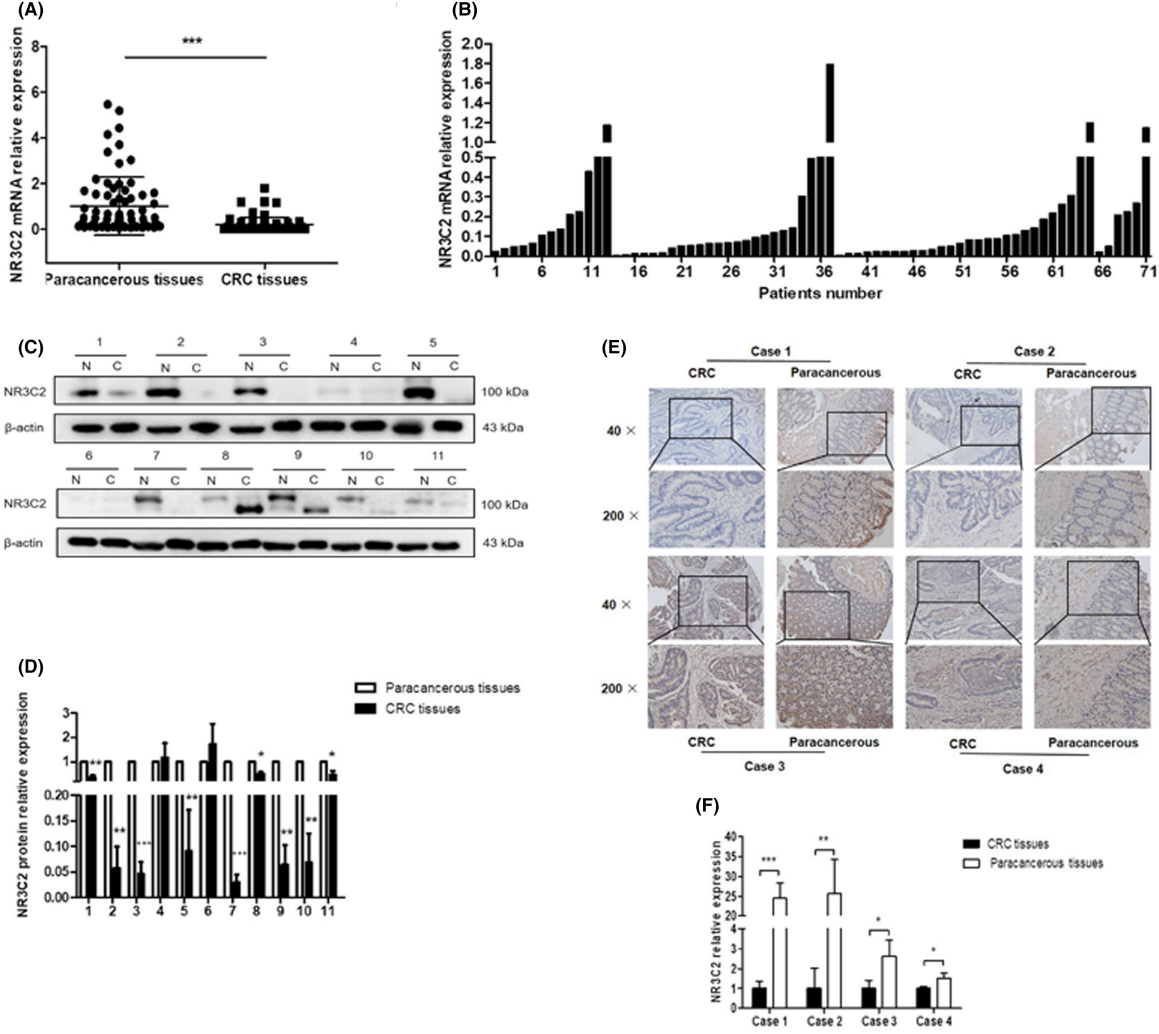
NR3C2 expression was significantly decreased in CRC tissues compared to paracancerous tissues. (A) The mRNA expression of NR3C2 in CRC tumours and paracancerous tissues in 71 CRC patients. (B) The mRNA expressions of NR3C2 in CRC tissues of patients with different clinic stages. (1–13: stage I; 14–37: stage II; 38–65: stage III; 66–71: stage IV). (C, D) The protein expression of NR3C2 in CRC tissues compared to paracancerous tissues of 11 CRC patients was detected by western blotting. (N: paracancerous tissues; C: colorectal cancer). (E, F) The expression of NR3C2 in four pairs of CRC tumour and paracancerous tissues was evaluated by IHC. *p < 0.05, **p < 0.01 and ***p < 0.001. CRC, colorectal cancer; IHC, immunohistochemitry.

The expression of NR3C2 in CRC tissues and paracancerous tissues of 71 CRC patients was determined by real‐time PCR and western blotting. As shown in Figure 2A, the mRNA levels of NR3C2 in CRC tumours were significantly decreased compared to matched paracancerous tissues. The downregulation of NR3C2 was especially obvious in stage II and III CRC tissues (Figure 2B). Moreover, the decreased expression of NR3C2 was significantly correlated with advanced stages and distant metastasis of CRC patients (Table 1). IHC staining results showed that the protein levels of NR3C2 were also decreased in CRC tumours compared to paracancerous tissues (Figure 2E,F). The western blotting analysis further confirmed the IHC results (Figure 2C,D).

**TABLE 1:** The relationship of clinical pathological features and *NR3C2* expression status in 71 CRC patients.

Clinical characteristics	*N* (Total %)	*NR3C2* relative expression		*p* Value
		Upregulation	Downregulation	
		*N* (total %) and (subject %)	*N* (total %) and (subject %)	
Gender				
Male	38 (53.52)	10 (14.08) (26.32)	28 (39.44) (73.68)	0.841
Female	33 (46.48)	8 (11.27) (24.24)	25 (35.21) (75.76)	
Age (years)				
≥56	49 (69.01)	14 (19.72) (28.57)	35 (49.30) (71.43)	0.352
<56	22 (30.99)	4 (5.63) (18.18)	18 (25.35) (81.82)	
Size (cm)				
≥5	18 (25.35)	4 (5.63) (22.22)	14 (19.72) (77.78)	0.724
<5	53 (74.65)	14 (19.72) (26.42)	39 (14.08) (73.58)	
Location				
Left	57 (80.28)	16 (22.54) (28.07)	41 (57.75) (71.93)	0.288
Right	14 (19.72)	2 (2.82) (14.29)	12 (16.90) (85.71)	
Node status				
N0	38 (53.52)	10 (14.08) (26.32)	28 (39.44) (73.68)	0.841
N1–2	33 (46.48)	8 (11.27) (24.24)	25 (35.21) (75.76)	
Distant metastasis				
M0	65 (91.55)	14 (19.72) (21.54)	51 (71.83) (78.46)	0.015*
M1	6 (8.45)	4 (5.63) (66.67)	2 (2.82) (33.33)	
Clinical stage				
I	13 (18.31)	5 (7.04) (38.64)	8 (11.27) (61.54)	0.039*
II	24 (33.80)	4 (5.63) (16.67)	20 (28.17) (83.33)	
III	28 (39.44)	5 (7.04) (17.86)	23 (32.39) (82.14)	
IV	6 (8.45)	4 (5.63) (66.67)	2 (2.82) (33.33)	

* *p* < 0.05.

### 3.3 Determination of the efficiency of NR3C2 overexpression and knock‐down

**FIGURE 3:**
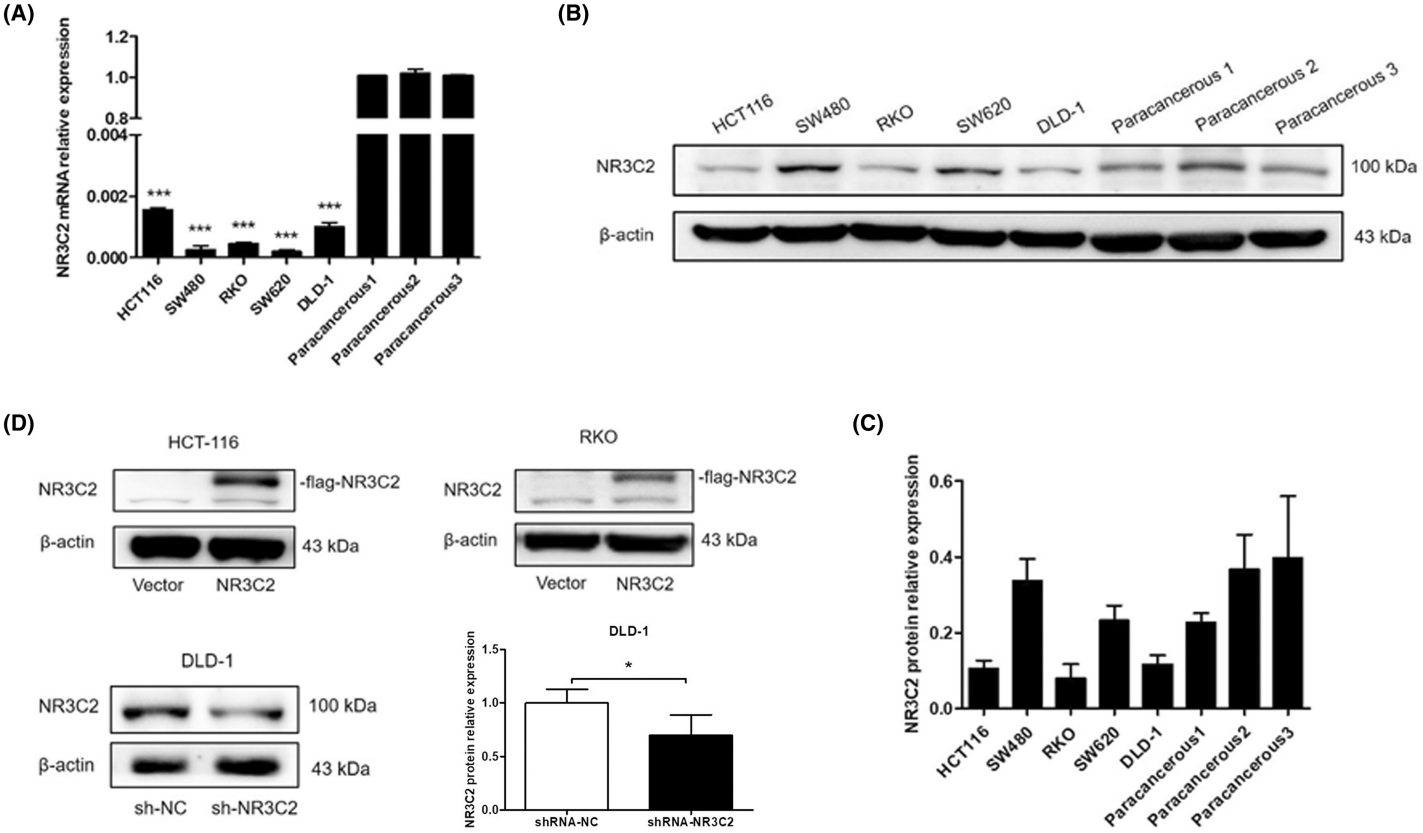
The expression of NR3C2 was downregulated in CRC cells. (A) The relative mRNA expression of NR3C2 in five CRC cell lines, HCT116, DLD‐1, RKO, SW480, SW620 and paracancerous tissues of three CRC patients (paracancerous 1, 2 and 3). (B,C) The protein expression of NR3C2 in five CRC cell lines, HCT116, DLD‐1, RKO, SW480, SW620 and paracancerous tissues of three CRC patients (paracancerous 1, 2 and 3). (D) The expression of NR3C2 in HCT116, RKO and DLD‐1 cell lines was overexpressed, and knocked down, respectively, and confirmed. *p < 0.05 and ***p < 0.001. CRC, colorectal cancer.

The expression of NR3C2 in human colon cancer cell lines including HCT116, DLD‐1, RKO, SW480 and SW620 cells was detected. As shown in Figure 3A, the mRNA expression of NR3C2 was significantly decreased in all five colon cancer cell lines compared with the paracancerous tissues. Besides, the protein expression of NR3C2 was lower in HCT116, RKO and DLD‐1 cells than in paracancerous tissues (Figure 3B,C). Among the five colon cancer cell lines, RKO and HCT116 cells exhibited a relatively lower expression of NR3C2, while DLD‐1 cells exhibited a relatively higher expression of NR3C2. Therefore, HCT116 and RKO cells were chosen to construct the NR3C2 overexpression cell lines (HCT116/NR3C2; RKO/NR3C2), with empty vector‐transfected cells (HCT116/Vector; RKO/Vector) as negative control (Figure 3D). Besides, DLD‐1 cells were chosen to construct an NR3C2 knock‐down cell line (DLD‐1/sh‐NR3C2), with control shRNA‐transfected cells (DLD‐1/sh‐NC) used as control (Figure 3D).

### 3.4 NR3C2 inhibited the proliferation and induced cell cycle arrest of CRC cells

**FIGURE 4:**
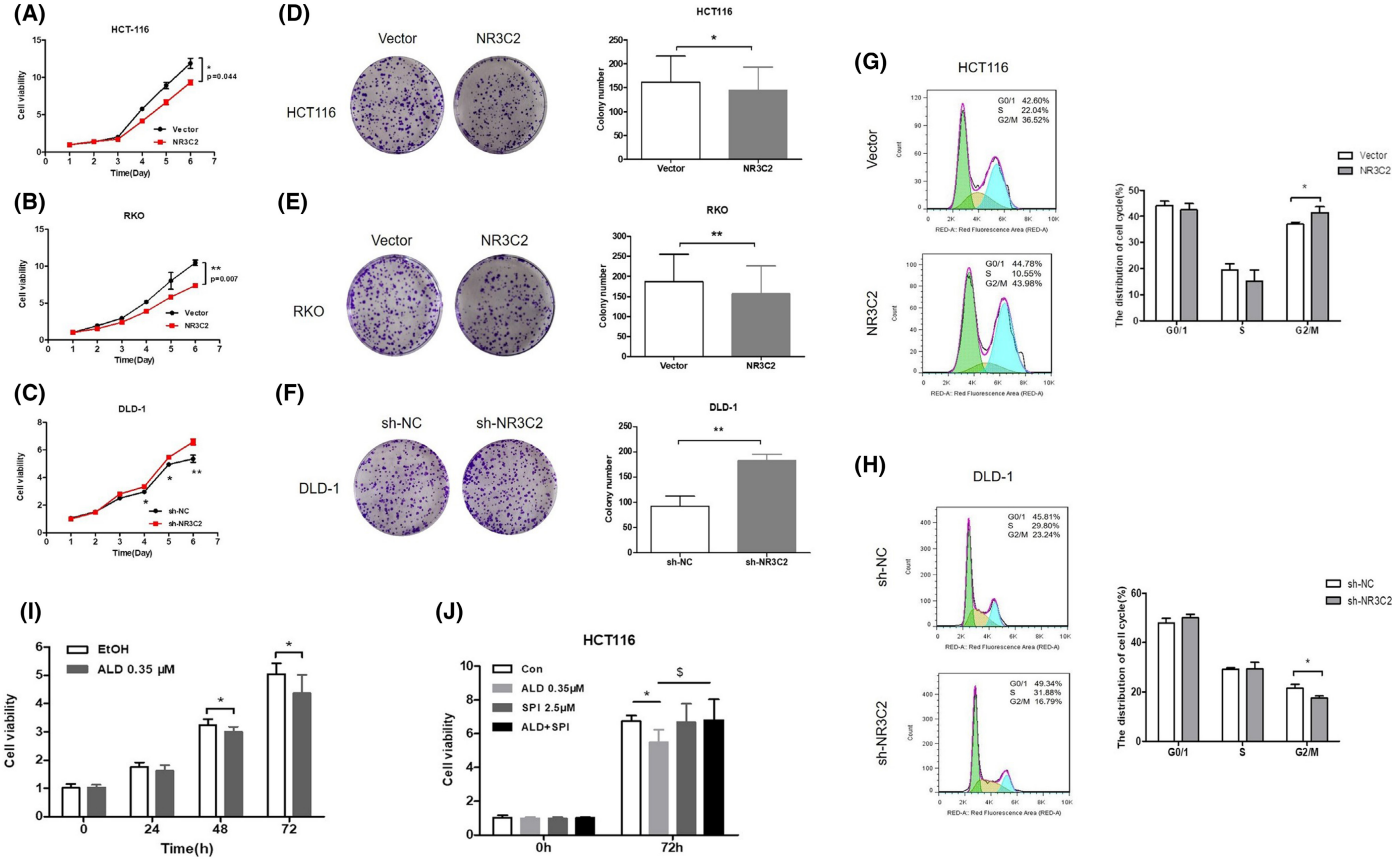
Overexpression of NR3C2 inhibited the proliferation and colony formation, and induced cell cycle arrest in CRC cells. (A–C) The influence of overexpressing or knocking down NR3C2 on the proliferation of CRC cells was determined by MTT assay. (D–F) The influence of overexpressing or knocking down NR3C2 on the capacity to form colonies of CRC cells was determined by a colony‐forming assay. (G, H) The influence of overexpressing or knocking down NR3C2 on the cell cycle arrest of CRC cells was determined by flow cytometry. (I) The viability of HCT116 cells treated with Ald was measured by MTT assay. (J) The viability of HCT116 cells treated with Ald, SPI or their combination was measured by MTT assay. *p < 0.05, **p < 0.01, ***p < 0.001 and ^$^ *p* < 0.05. Ald, aldosterone; CRC, colorectal cancer; MTT, 4,5‐dimethyl‐2‐thiazolyl)‐2,5‐diphenyl‐2‐H‐tetrazolium bromide; SPI, spirolactone.

As shown in Figure 4A–F, the proliferation and colony formation were inhibited when overexpressing NR3C2 in RKO and HCT116 cells. Moreover, knock‐down NR3C2 promoted the proliferation and colony formation of DLD‐1 cells. In addition, we found that HCT116 cells overexpressing NR3C2 presented smaller volumes than HCT116/Vector cells in the subcutaneous tissue of nude mice (Figure S1). Flow cytometry analysis showed that compared to the Vector group, the percentage of cells at the G2/M phase was significantly increased after overexpressing NR3C2 (Figure 4G). In contrast, knocking down NR3C2 leads to a decreased percentage of cells in the G2/M phase (Figure 4H).

As the ligand of hMR, increased Ald level with the treatment of cediranib might be predictive of overall survival in patients with non‐small‐cell lung cancer.^20^ So, we tried to explore whether Ald could attenuate the proliferation of CRC cells. As shown in Figure 4I, the proliferation of HCT116 cells was significantly suppressed after treatment with 0.35 μM Ald for 72 h. Moreover, the anti‐proliferation effects of Ald were blocked by SPI, the antagonist of hMR (Figure 4J).

### 3.5 NR3C2 regulated glucose metabolism by inhibiting the expression of hexokinase 2 (HK2) and lactate dehydrogenase A (LDHA)

**FIGURE 5:**
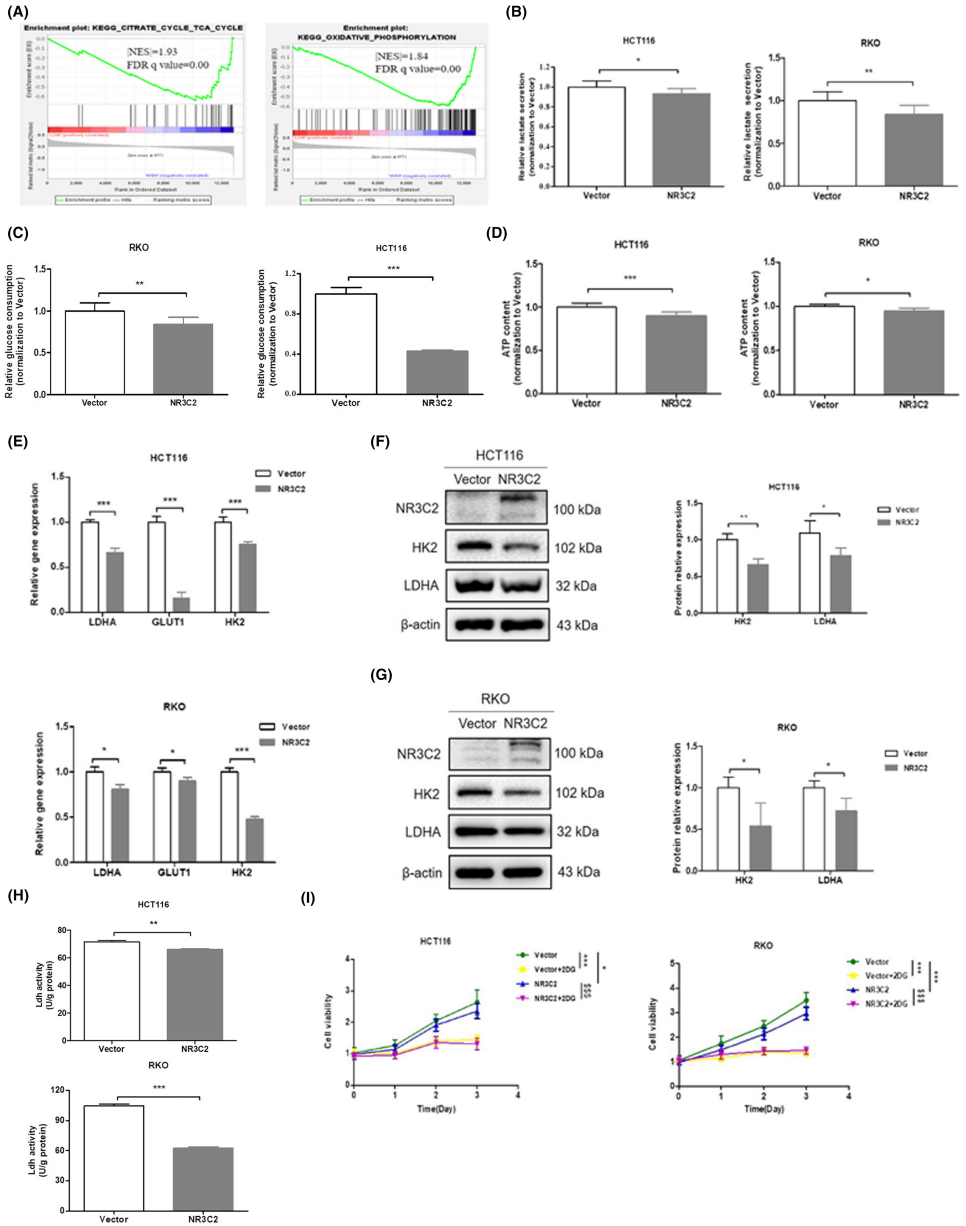
NR3C2 inhibited glucose metabolism in CRC cells via HK2 and LDHA. (A) The two GSEA profiles for the altered genes data by comparing the NR3C2 high and low expression sample groups from the GEO data set. (B) Overexpressing NR3C2 decreased lactate secretion in HCT116 and RKO cells. (C) Overexpressing NR3C2 decreased glucose consumption in HCT116 and RKO cells. (D) Overexpressing NR3C2 decreased ATP content in HCT116 and RKO cells. (E) Overexpressing NR3C2 inhibited the mRNA expression of LDHA, HK2 and GLUT1 in HCT116 and RKO cells. (F, G) Overexpressing NR3C2 inhibited the protein expression of LDHA and HK2 in HCT116 and RKO cells. (H) Overexpressing NR3C2 inhibited the Ldh activity in HCT116 and RKO cells. (I) The effect of 2‐DG on the growth of HCT116 and RKO cells. *p < 0.05, **p < 0.01 and ***p < 0.001versus Vector group; ^$$$^ *p* < 0.001 versus NR3C2 group. 2‐DG, 2‐Deoxy‐d‐glucose; CRC, colorectal cancer; GEO, gene expression omnibus; GLUT1, Glucose transporter‐1; GSEA, gene set enrichment analysis; HK2, hexokinase 2; Ldh, Lactate dehydrogenase; LDHA, lactate dehydrogenase.

To further elucidate the underlying mechanisms of NR3C2 in CRC, we used GSEA to analyse the set of genes altered by NR3C2 in human CRC samples from the GEO data set (GSE41258, *n* = 94 in the high expression group and *n* = 92 in the low expression group). As shown in Figure 5A, two pathways were correlated with high expression of NR3C2, citrate cycle and oxidative phosphorylation. A high rate of glucose catabolism into lactate was the most ubiquitous metabolic phenotype across cancer cells.^21^ In our results, glucose consumption, lactate secretion and ATP content in HCT116 and RKO cells were all reduced when overexpressing NR3C2 (Figure 5B–D). Besides, overexpressing NR3C2 significantly decreased the mRNA and protein levels of LDHA and HK2 which were the key enzymes of glucose metabolism (Figure 5E–G). Lactate dehydrogenase (Ldh) turns pyruvate into lactate during aerobic glycolysis. Inhibiting Ldh activity decreases the production of lactate and impairs cell growth.^22^ As shown in Figure 5H, overexpression of NR3C2 effectively inhibited the activity of Ldh. The inhibitor of glycolysis 2‐DG significantly inhibited the growth of Vector and NR3C2 cells in HCT116 and RKO, but there was no significant difference between the two groups (Figure 5I). Taken together, the inhibition of CRC cell growth by NR3C2 was associated with the suppression of the glucose metabolism.

### 3.6 NR3C2 influenced CRC cell growth via AMPK

**FIGURE 6:**
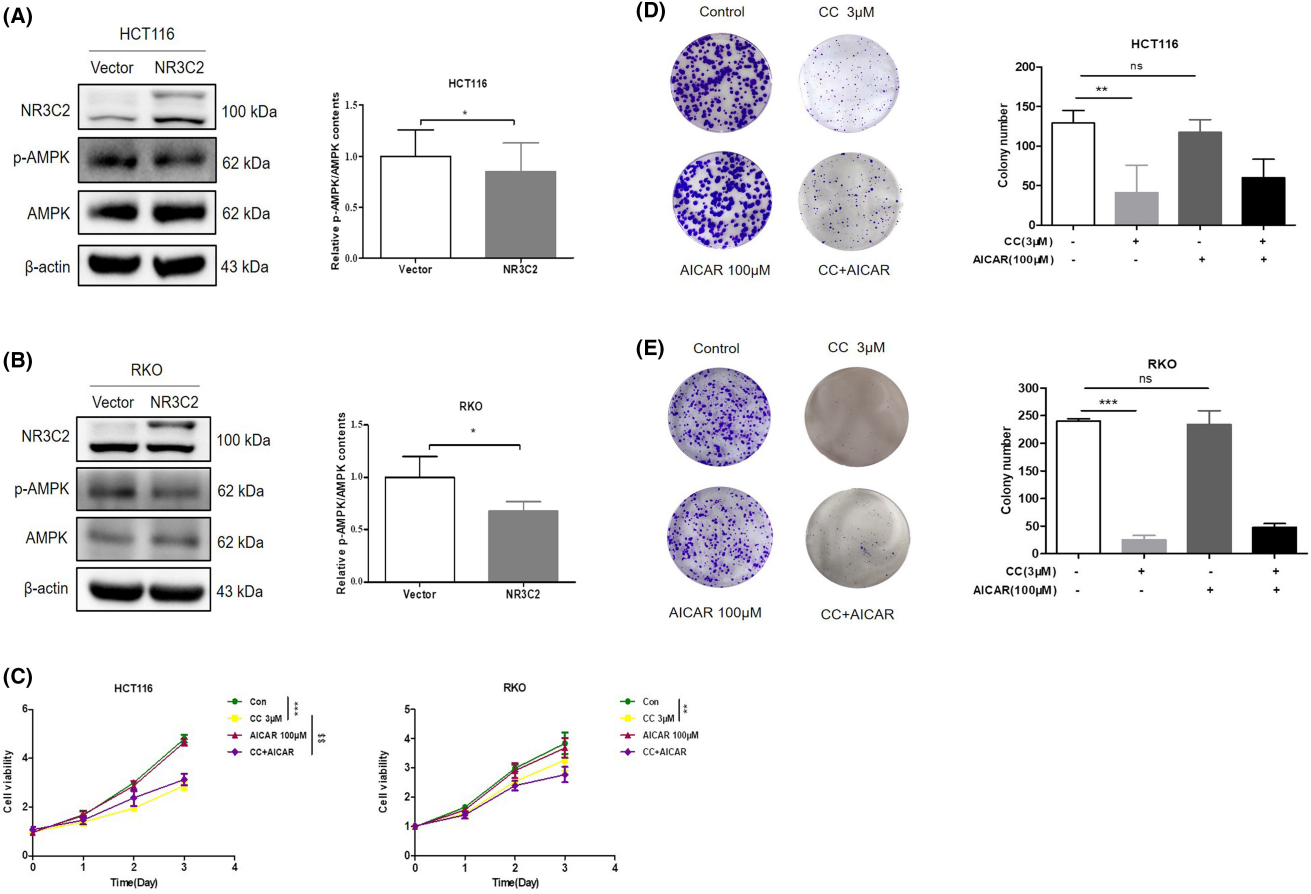
NR3C2 inhibited the AMPK phosphorylation of CRC cells. (A) NR3C2 inhibited the phosphorylation of AMPK in HCT116 cells. (B) NR3C2 inhibited the phosphorylation of AMPK in RKO cells. (C) Effects of AICAR and CC on the growth of HCT116 and RKO cells. (D) Effects of AICAR and CC on the colony formation of HCT116 cells. (E) Effects of AICAR and CC on the colony formation of RKO cells. *p < 0.05 versus the Vector group; **p < 0.01 and ***p < 0.001 versus con group, ^$$^ *p* < 0.01 versus the CC group; ns: no significant difference. AMPK, adenosine 5′‐monophosphate (AMP)‐activated protein kinase; CC, Compound C; con, Control.

**FIGURE 7:**
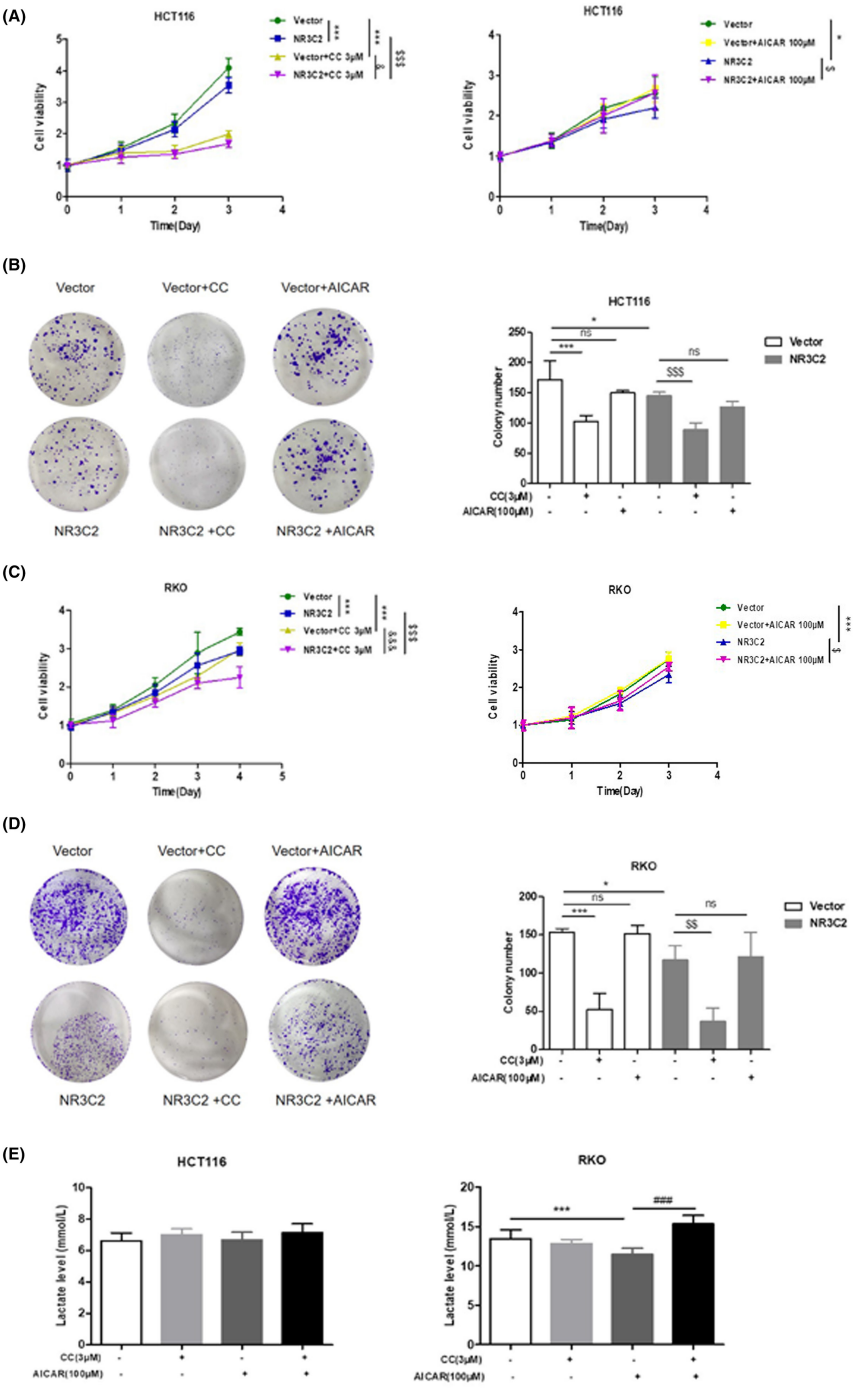
NR3C2 inhibited the proliferation of HCT116 and RKO cells partly through AMPK. (A) Effects of CC and AICAR on the proliferation of HCT116/NR3C2 and HCT116/Vector cells. (B) Effects of CC and AICAR on the colony formation of HCT116/NR3C2 and HCT116/Vector cells. (C) Effects of CC and AICAR on the proliferation of RKO/NR3C2 and RKO/Vector cells. (D) Effects of CC and AICAR on the colony formation of RKO/NR3C2 and RKO/Vector cells. (E) Effect of AICAR and CC on the lactate secretion of HCT116 and RKO cells. *p < 0.05 and ***p < 0.001 versus the Vector group; ^$^ *p* < 0.05, ^$$^ *p* < 0.01 and ^$$$^ *p* < 0.001 versus the NR3C2 group; ^&^ *p* < 0.05, ^&&&^ *p* < 0.001 versus the Vector+CC group; ^###^ *p* < 0.001 versus the AICAR group; ns: no significant difference.

AMPK is the energetic sensor maintaining cellular energy homeostasis. In cancer cells, AMPK may play different roles depending on the tumour microenvironment.^13^ Overexpression of NR3C2 significantly inhibited AMPK phosphorylation in RKO and HCT116 cells (Figure 6A,B). As shown in Figure 6C–E, CRC cell growth and colony formation were significantly inhibited by the AMPK inhibitor CC. Although adding AMPK agonist AICAR alone did not significantly promote cell growth and colony formation, combining AMPK agonist AICAR with inhibitor partially reversed the inhibitory effect of CC. Therefore, we wanted to further explore whether NR3C2 regulates CRC cell proliferation through AMPK. Our results showed that CC has a stronger inhibitory effect on HCT116/NR3C2 and RKO/NR3C2 cells compared to HCT116/Vector and RKO/Vector cells (Figure 7A,C). The inhibitory effect of CC on HCT116/NR3C2 and RKO/NR3C2 cell clone formation is consistent with cell proliferation results (Figure 7B,D). Furthermore, AICAR significantly increased cell growth in the HCT116/NR3C2 and RKO/NR3C2 cells, but not in the HCT116/Vector and RKO/Vector cells (Figure 7A,C). However, AICAR did not significantly enhance the colony formation of HCT116/NR3C2 and RKO/NR3C2 cells (Figure 7B,D). Next, we try to determine if NR3C2 suppresses glycolysis through AMPK. As shown in Figure 7E, CC alone did not significantly increase lactate secretion, while the combination of AICAR and CC reversed the inhibitory effect of AICAR on lactate secretion in RKO. However, lactate secretion was not affected by changes in AMPK activity in HCT116 cells. Collectively, NR3C2 inhibits CRC cell proliferation via AMPK, but the regulation of glycolysis is independent of AMPK.

## 4 DISCUSSION

Through bioinformatics analysis for several databases, 72 DEGs were identified. Among them, NR3C2 was demonstrated to be correlated with poor prognosis and tumour staging of CRC, which may serve as a therapeutic target. One bioinformatic study revealed that NR3C2 was downregulated in six types of cancers including bladder, breast, colon, head and neck, liver and prostate.^23^ Besides, NR3C2 was associated with CRC overall survival and tumour stage categorized into stages I and II but not with other pathophysiological characteristics in a study including 30 CRC patients.^9^ Our results in 71 clinical samples were similar to the previous study, which validated our bioinformatics analysis above. We found that NR3C2 was downregulated in CRC and correlated with not only tumour stages but also distant metastasis. However, Di Fabio et al. found that the expression of NR3C2 was not associated with tumour stage or differentiation in a study of 48 CRC patient tissues.^8^ But NR3C2 was significantly downregulated in three VEGFR‐2 high expressions CRC tissues: right‐sided, poorly differentiated and lymph node metastatic carcinomas.^8^

NR3C2 was reported to inhibit the proliferation of renal cancer cells in vitro and in vivo.^7^ As the target gene of miR‐766, NR3C2 participated in the β‐catenin signalling pathway to inhibit the proliferation and metastasis of liver cancer cells.^6^ Moreover, overexpression of NR3C2 inhibited the proliferation of LoVo cells through the AKT/ERK pathway.^10^ In our study, lentivirus was used to overexpress or knock‐down NR3C2 in HCT116, RKO and DLD‐1 cells. Our results further validated that NR3C2 inhibited the proliferation and induced cell cycle arrest of CRC cells. And more importantly, we found that overexpression of NR3C2 inhibited the proliferation of CRC cells partially by regulating glucose metabolism and inhibiting AMPK activity. Normally, cells convert most of the glucose they uptake to pyruvate through glycolysis. As a fuel, pyruvate participates in the tricarboxylic acid (TCA) cycle and oxidative phosphorylation to generate a large number of energies in the form of ATP.^21^ To maintain their energy and anabolic demands, cancer cells consume more glucose than normal cells and tend to convert much of the pyruvate into lactate, which is then excreted into the extracellular medium.^21^ At the beginning of glycolysis, glucose is phosphorylated to glucose‐6‐phosphate by hexokinase. As a member of hexokinase, HK2 is located on the outer membrane of mitochondria and has been shown to be upregulated in cancers.^24^ LDHA is one of the most important isozymes of lactate dehydrogenases, which is involved in the conversion of pyruvate to lactate. Several studies have indicated that LDHA is abnormally expressed in a variety of solid tumour cells.^25^ Researchers found that CRC patients with both low LDHA expression and mismatch repair deficiency exhibited better disease‐free survival compared with others.^26^ Studies over the past few years indicated that altered activation of hMR which is encoded by NR3C2 is associated with metabolic and cardiovascular dysfunctions.^16^ However, the effect of NR3C2 on glucose metabolism in CRC cells has not been reported. Our study found that NR3C2 overexpression reduced glucose consumption, lactate production and ATP production by regulating the expression of HK2 and LDHA. In addition to HK2 and LDHA, some researchers discovered that NR3C2 inhibits cellular aerobic glycolysis by regulating PKLR, an isozyme of pyruvate kinase, in hepatocellular carcinoma cells.^15^

AMPK is a well‐known energy sensor that plays important role in sustaining energy homeostasis and is implicated in the development of cancer. The role of AMPK in tumour cells is still controversial. Many studies support the tumour‐suppressive role of AMPK, but emerging evidence suggests that under certain circumstances cancer cells utilize AMPK to gain a growth advantage.^27^ Both AMPK and pAMPK expressions were found to be higher in cancerous tissues than in paraneoplastic tissues in CRC and elevated AMPK was associated with lymph node and distant metastases.^12^, ^13^ Our results found that NR3C2 significantly inhibited the phosphorylation of AMPK. Results of cell growth and clone formation by overexpression of NR3C2 in combination with AMPK agonists and inhibitors inferred that NR3C2 affects CRC cell growth partly through AMPK. Inhibiting AMPK was reported to increase the lactate secretion in HT‐29 cells while activating AMPK decreased the lactate secretion, which was opposite to the effect of AMPK on cell growth.^12^ However, pancreatic cancer patients with high AMPK expression have a worse prognosis. Inhibiting AMPK significantly reduces cellular lactate secretion, glucose consumption, ATP production, HK2 and PKM2 expression.^28^ Our findings agree with Xiao et al.^12^ that lactate secretion is reduced after AMPK activation in RKO cells. Additionally, glucose activates AMPK expression to selectively induce EP300 which then promotes differentiation towards β‐catenin and promotes cell proliferation in colorectal cancer finally.^29^ Therefore, we speculate that NR3C2 might influence β‐catenin expression through AMPK to regulate CRC cell proliferation which remains to be further elucidated.

The hMR has two physiological ligands, Ald and cortisol. hMR is co‐expressed with 11βHSD2 in polarized tight epithelia and involved in aldosterone‐dependent transepithelial sodium transport.^30^ Aldosterone and cortisol in combination inhibit the growth of breast cancer cells expressing MR and 11βHSD2.^31^, ^32^ Besides, Ald attenuated the proliferation and induced apoptosis of hepatocellular carcinoma cells, which was reversed by SPI.^15^ Tiberio et al. reported that hMR activation by aldosterone induces a significant decrease in VEGFA mRNA expression in HCT116 cells under both normal and hypoxic environments, which was partially blocked by SPI.^9^ Therefore, we hypothesized that NR3C2 may serve as a potential pharmacological target of CRC. Our results showed that Ald inhibited the proliferation of HCT116 cells, which was reversed by SPI. Contrary to our results, Ald was reported to promote the survival and proliferation of renal carcinoma cells co‐expressing hMR and 11βHSD2,^33^, ^34^ which may be due to the expression and the roles of NR3C2 varied in organs. Several cofactors have been found to interact and regulate the activity and selectivity of hMR.^17^

In our study, we have unveiled and validated that NR3C2 suppresses CRC tumorigenesis and correlated with the advanced stages and distant metastasis of CRC. To the best of our knowledge, it is the first report on the relationship between NR3C2 and glucose metabolism and AMPK in CRC cells.

## AUTHOR CONTRIBUTIONS

**Hui Liu:** Formal analysis (equal); investigation (lead); methodology (lead); writing – original draft (lead). **Wenqi Lei:** Investigation (supporting). **Zhigui Li:** Investigation (supporting). **Xiaodong Wang:** Supervision (equal). **Liming Zhou:** Supervision (lead).

## CONFLICT OF INTEREST STATEMENT

The authors confirm that there are no conflicts of interest.

## CONSENT

Written informed consent was obtained from patients before sampling.

## Supporting information


Appendix S1

## Data Availability

The data sets generated during this study are available from the corresponding author upon reasonable request.
