# Supplementary material for: NR3C2 inhibits the proliferation of colorectal cancer via regulating glucose metabolism and phosphorylating AMPK
Source: J Cell Mol Med. 2023 Mar 23;27(8):1069–82. doi: 10.1111/jcmm.17706 (PMC10098300; doi:10.1111/jcmm.17706)
Supplement: Supplementary file 1 — Appendix S1 [file JCMM-27-1069-s001.docx]

**Supplementary data**

**Supplemental experimental procedures:**

**In vivo experiments**

Eight 5-week-old female BALB/C-Nude mice (Gempharmatech, Chengdu, China) were used for the xenograft experiment. Mice were randomly assigned into the Vector group and NR3C2 group. HCT116/Vector and HCT116/NR3C2 cells were resuspended with a basement membrane-like matrix (3432-005-01, R&D Systems) and inoculated 1×10^7^ cells/100μL/mice subcutaneously. The subcutaneous tumor volume in nude mice was measured and calculated according to the following formula: V = 0.5 × lengths × width^2^. Two weeks later, the nude mice were sacrificed and the tumors were collected. This experiment was in accordance with the National Institutes of Health Guide for the Care and Use of Laboratory Animals and was approved by the Ethics Committee of West China Hospital (K2022005).

**Supplemental Figure**

**Figure s1.**


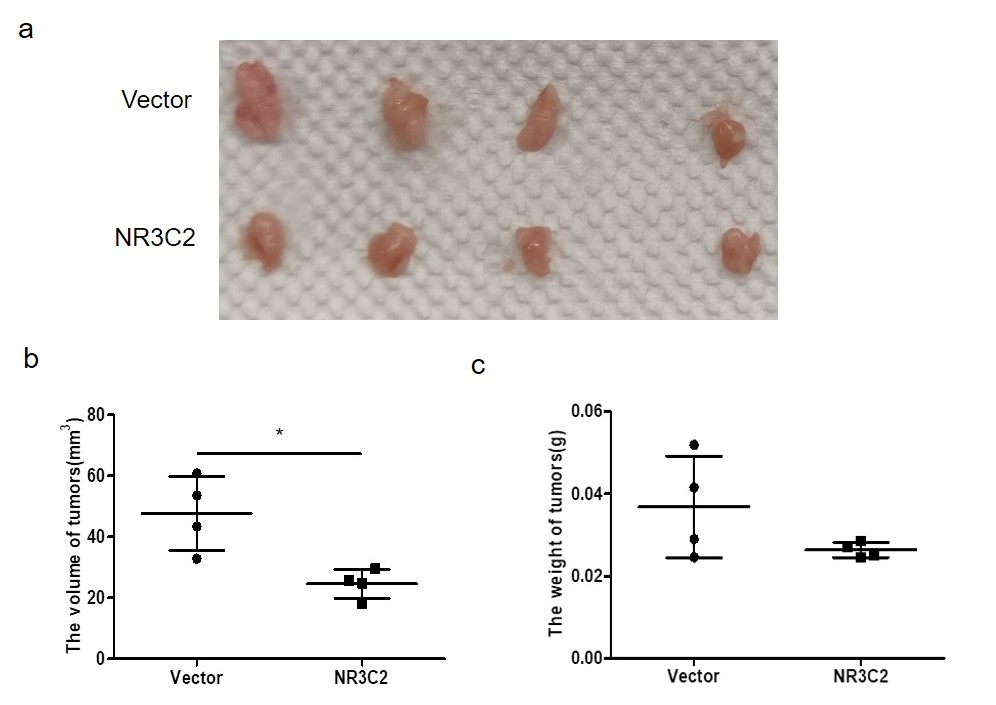


**Supplemental Figure Legends:**

**Figure S1.** Over-expressing NR3C2 inhibited tumor growth *in vivo*. **a** HCT116/Vector and HCT116/NR3C2 cells were subcutaneously injected into female BALB/C-Nude mice (n=4). Images of tumors in nude mice exhibition. **b** The tumor volume(mm^3^) of xenograft. **c** The tumor weights (g) of xenografts. *p < 0.05
